# Editorial: Possible applications of neuroaesthetics to normal and pathological behaviour

**DOI:** 10.3389/fnins.2023.1225308

**Published:** 2023-07-14

**Authors:** Pietro Sarasso, Gianni Francesetti, Felix Schoeller

**Affiliations:** ^1^Brain Plasticity and Behaviour Changes Research Group, Department of Psychology, University of Turin, Turin, Italy; ^2^International Institute for Gestalt Therapy and Psychopathology, Turin, Italy; ^3^Department of Psychology, University of Turin, Turin, Italy; ^4^Institute for Advanced Consciousness Studies, Santa Monica, CA, United States; ^5^Massachusetts Institute of Technology, Cambridge, MA, United States

**Keywords:** aesthetics, neuroaesthetics, embodied aesthetics, artistic interventions, psychopathology, interoceptive technology, learning, aesthetic chills

## Highlights

- Aesthetic pleasure is a fundamental aspect of human perception, extending beyond the appreciation of artworks or superficial visual stimuli.
- Neuroaesthetics has potential therapeutic applications and can be used to induce behavioral change.
- Curiosity and beauty perception are interconnected, and aesthetic experiences have implications for various areas such as knowledge acquisition, motivation, memory formation, and pain perception.
- Incorporating artistic interventions into treatment plans for neurological conditions can improve overall wellbeing and quality of life.
- Embodied aesthetics explores the relationship between interoception, emotional experiences, and neuropsychiatric disorders, and has potential implications for the restoration of interoceptive inference and treatment of conditions like depression.

## 1. Introduction

In recent years, a perspective on aesthetics has resurfaced as an essential evolutionary dimension of human learning, permeating basic brain functions such as cognition, perception, and emotion and indispensable to behavioral change (Biederman and Vessel, 2006; Schaeffer, 2013; Schoeller and Perlovsky, 2016; Sarasso et al., 2019; Wassiliwizky and Menninghaus, 2021; Kenett et al., 2023; Sarasso et al.). This view circles back to the original vision of Baumgarten in 1735, who defined aesthetics as the “science of sensory knowledge directed toward beauty” (Colin McQuillan, 2021), beyond the study of artistic production and consumption (Berlyne, 1973; Sarasso et al., 2020). As mentioned by Magsamen et al. in this volume, aesthetics derives from the Greek word “aisthēsis,” which means “sensation,” “perception,” (as in “anesthetics”), the very foundation of human experience. Aesthetic pleasure is not confined to the appreciation of artworks or superficial visual stimuli; rather, it is a fundamental requirement for human perception (Schoeller et al., 2018b) and a necessary condition to virtually every human activity (Vessel et al., 2012, 2013; Belfi et al., 2019), including speech perception and decision-making (Perlovsky and Schoeller, 2019). In other words, it is “always on” (Wassiliwizky and Menninghaus, 2021).

## 2. Neurasthenics as a promising field for therapeutic applications

This Research Topic seeks to answer the central research question of what endogenous mechanisms and exogenous factors make humans more or less sensitive to sensory novelty, exploring the potential applications of neurasthenics to psychopathology. The ultimate aim is to examine how aesthetic experiences can be used therapeutically to foster meaningful, desirable, behavioral change in a wide range of settings inducing psychedelic-assisted psychotherapy (Strickland et al., 2021). As the articles in this Research Topic show, the neo-Baumgartian perspective is increasingly pervading empirical aesthetics. As reviewed by Magsamen et al. in this Research Topic, neuroaesthetics now encompasses a wide array of studies and initiatives at the intersection of arts and aesthetics with health, wellbeing, and learning. This creates a pressing need for a new framework to support and potentially accelerate neurasthenic's research and translation.

## 3. Curiosity and beauty perception

Scientists are beginning to explore the link between curiosity and beauty perception (Gottlieb et al., 2013; Schoeller, 2016; Tik et al., 2018; Van de Cruys et al., 2021), knowledge-acquisition processes in general (Schoeller and Perlovsky, 2016; Grossberg and Zajac, 2017; Schwartenbeck et al., 2019), the tuning of the sensory system to perceptual (Brielmann and Dayan, 2021) and social (Sarasso et al., 2021b) experiences, intrinsic motivation in educational activities (Howard et al., 2020), memory formation (Sarasso et al., 2021a), pain perception (Mitchell et al., 2008), neurodegeneration (Kliem et al.), diagnostic processes (Spee et al.), therapeutic change (Higginson et al., 2011; Connolly, 2021; Sarasso et al.), and many other topics. Beyond art therapy, we begin to see how these insights can be applied to better understand both normal and pathological behavior, from psychopathology and brain damage to cognitive impairments associated with aging.

## 4. Implications for treatment

In this Research Topic, van Leeuven et al. provide a compelling example of the overlap between artistic and social brain function, which has significant practical implications for mental health and the care of individuals living with neurological conditions such as dementia. The use of artistic stimuli by Kliem et al. to investigate the stability of preferences in pathological aging also sheds light on the potential dual path of cognitive deterioration in Alzheimer's disease and dementia, as suggested by recent studies like Felisberti's (Garrido, 2019; Felisberti, 2021). These findings underscore the importance of incorporating artistic interventions into treatment plans for neurological conditions to improve overall wellbeing and quality of life (Kliem et al.). The potential preservation of a pleasure and beauty pathway for perception and memory in pathological aging raises the possibility of a revolutionary shift in the design of rehabilitation protocols. If such a pathway exists, it could have profound implications for enhancing the quality of life and wellbeing of individuals with neurological conditions. Future research on this topic may provide valuable insights into the development of targeted interventions that leverage the power of aesthetics to support cognitive function in aging and neurological disease.

## 5. Embodied aesthetics

When the study of perceptual inference focuses on changes inside the body (i.e., interoception) we might refer to embodied aesthetics (Kirsch et al., 2016; Koch, 2017; Kühnapfel et al., 2023). This line of research is growingly interested in how interoceptive technologies might help to restore interoceptive inference in a variety of neuropsychiatric disorders (Riva et al., 2017, 2021; Riva, 2018; Schoeller et al., 2022a; Di Lernia and Riva, 2023), especially those characterized by a loss of embodied resonance such as depression (Fuchs and Koch, 2014). Based on an embodied model of emotion (Pezzulo, 2014), Schoeller et al., shed new light on this topic by showing that simulating the somatic markers of the aesthetic emotion of chills can influence valence and arousal perception (Schoeller et al., 2018a, 2019; Haar et al., 2020). In this Research Topic, Jain et al. present recent data collected using chillsDB (Schoeller et al., 2022b), an online catalog of chill-eliciting stimuli, showing that aesthetic chills induce a reliable shift in emotional valence and arousal (Jain et al.), extending prior results demonstrating the relevance of chills for depression (Schoeller et al., 2023). Aesthetic chills can cause an emotional breakthrough, reminiscent of some of the effects of the psychedelic experience (Roseman et al., 2019) and mitigating maladaptive cognitions such as shame and negative self-perception (Schoeller et al., 2023).

## 6. Conclusion

Theoretical models rooted in predictive processing and sensory inference offer an intriguing avenue of research in the field of neuroaesthetics (Schoeller and Perlovsky, 2015, 2016; Schoeller, 2016; Sarasso et al., 2020; Van de Cruys and Bervoets, 2022; Richard et al., 2023; Sarasso et al.; Spee et al.). According to these models, aesthetic pleasure arises from the fitting of predictive representations to sensory experiences, reflecting a neo-Kantian approach (Perlovsky, 2014; Schaeffer, 2015; Swanson, 2016). The application of such models to therapeutic activities aimed at changing rigid and repeating behavioral patterns (Spee et al.), such as psychotherapy (Sarasso et al.) and neurorehabilitation (Kliem et al.), has promising implications. Future research in this area could yield valuable insights into the underlying mechanisms of cognitive and emotional processing, and inform the development of targeted interventions to improve mental health and wellbeing.

## Author contributions

All authors listed have made a substantial, direct, and intellectual contribution to the work and approved it for publication.
